# HTPFA-Coated AlB_2_ with Enhanced Combustion Performance as a High-Energy Fuel

**DOI:** 10.3390/ma18071452

**Published:** 2025-03-25

**Authors:** Jiangfeng Wang, Wanjun Zhao, Chen Shen, Yapeng Ou, Qingjie Jiao

**Affiliations:** State Key Laboratory of Explosion Science and Technology, Beijing Institute of Technology, Beijing 100081, China; wangjiangfength@163.com (J.W.); shenchen1638@163.com (C.S.); ouyapeng@bit.edu.cn (Y.O.); jqj@bit.edu.cn (Q.J.)

**Keywords:** AlB_2_, functionalized fluoropolymer, ignition, combustion characteristic, preignition reaction

## Abstract

High-energy boron-based fuel aluminum-diboride (AlB_2_) has attracted much attention in the field of solid propellants. However, the low reactivity of AlB_2_ hindered its further application. In this study, highly reactive AlB_2_@hydroxyl-terminated perfluoropolyether alcohol (AlB_2_@HTPFA) composites with a core–shell structure were prepared by coating AlB_2_ with functionalized fluoropolymers by using a facile one-step in situ polymerization method. AlB_2_@HTPFA composites with varying polymer contents (0, 5, 10, and 15 wt%) were obtained. The in situ polymerization strategy enables precise control over the polymer coating thickness and interfacial interactions, which is critical for optimizing the reactivity and thermal stability of composites. The morphology and structure were characterized, and the microcore–shell structure of AlB_2_@HTPFA was obtained. Compared with raw AlB_2_, the combustion efficiency of coated fuel increased by 4.1%, 5.6%, and 7.5%, respectively, with varying polymer contents. Meanwhile, the reactivity of AlB_2_@HTPFA (5 wt%) was 0.65 MPa/s, which is ~1.5 times that of AlB_2_. Additionally, the ignition and combustion characteristics of AlB_2_@HTPFA were investigated by laser ignition experiments with potassium perchlorate (KP) as an oxidant. The results revealed that AlB_2_@HTPFA/KP composites showed a greatly reduced combustion duration compared to uncoated AlB_2_. The ignition and combustion enhancement mechanism of AlB_2_@HTPFA was proposed. During the ignition process, the existence of HTPFA can result in a pre-ignition reaction, thus raising its reaction activity. This work provided a promising candidate for high-energy fuel that can be used in energetic materials.

## 1. Introduction

AlB_2_, a boron-based compound, has attracted large amounts of interests for application in energetic materials due to its higher volumetric heat of 122–140 kJ/cm^3^, higher energy release rate, and higher combustion efficiency [1,2,3,4,5] compared with one of boron [6,7,8]. Moreover, AlB_2_ could reduce the ignition delay of high-energy formulations by 2–2.5 times [9,10]. However, during the combustion of AlB_2_, B_2_O_3_ with a low melting point (450 °C) and a high boiling point (1860 °C) [11] emerged as an impervious liquid film on the surface, preventing AlB_2_ contacting with oxygen and thus reducing combustion efficiency in the initial stages. It has been reported that the combustion characteristics of boron-containing fuels could be improved by changing their surface chemicals [12,13,14]. To the best of our knowledge, there were few studies about the coating of AlB_2_. Currently, fluorine-containing organic compounds (FCOCs), particularly polymers rich in fluorine, are interesting candidates for being employed as energetic additives [15,16]. In the realm of energy materials, FCOC-containing substable hybrid composites have drawn more and more attention recently. According to the reported literature [17,18,19,20], FCOCs were frequently added to materials containing aluminum to enhance the ignition and combustion performance. It can be attributed to the fact that fluoropolymers, acting as oxidizing agents, could stimulate surface exothermic reactions between the alumina shells and fluorine, thus enhancing ignition and combustion [21,22,23]. Wang et al. [24] conducted a comparative study of the ignition and combustion properties of Al-PVDF, Al-Viton (hexafluoropropylene and vinylidene fluoride), and Al-THV (tetrafluoroethylene, hexafluoropropylene, and vinylidene fluoride), revealing that the content of fluorine played an important role in determining the combustion characteristics of energetic composites. In contrast to coating B with metal and organic chemicals, fluorine-containing materials could also be utilized [25]. Fluorinated modification materials for boron-based fuels primarily include metal fluorides, fluorinated graphene, and fluoropolymers. Current studies on fluoropolymer coatings for boron-based fuels, including trifluoroethylene-HFP copolymer (Viton A) [26], trifluoroethylene-HFP copolymer (THF) [27], polyvinylidene fluoride (PVDF) [28], and polytetrafluoroethylene (PTFE) [29], demonstrate their capacity to reduce ignition delay, improve combustion duration, and enhance combustion efficiency. This performance enhancement arises partly from the fluorination reaction where B_2_O_3_ reacts with fluorine to form BF_3_ releasing 188.99 kcal/m^3^ [30,31,32]—which promotes boron oxidation kinetics [28,33], thereby enabling fluorine-containing polymers to optimize boron combustion. However, there were insufficient data stating the mechanism of the effect of fluorinated oxides on the energetic behavior of AlB_2_.

Fluoropolymers could be introduced into the system through chemical and physical methods. Viton A, THF, and PVDF are typically coated via recrystallization methods, whereas PTFE coatings are prepared through high-energy ball milling combined with recrystallization. Beyond these approaches, electrospinning [34,35] represents another common yet relatively complex fabrication process for fluoropolymer coatings. Usually, linking organic fluorine to the molecular network structure with chemical reaction could be clarified as the chemical method, for instance, end-hydroxy fluoroelastomer cured with isocyanate, end-carboxy fluoroelastomer cured with epoxy resin [36], and the fluorine modification of adhesive followed by curing agent reaction [37,38,39]. Considering the high fluorine content, inertia, and liquid state at room temperature, HTPFA was chosen as the fluorine-containing organic substance for the study of AlB_2_ [40,41]. Additionally, the hydroxyl group on the alcohol can be grafted into the adhesive network by reacting with the isocyanate group in the curing agent, improving the stability [42,43]. Notably, this study pioneers the selection of HTPFA as a fluoropolymer for coating AlB_2_ via a one-step in situ polymerization method. To the best of our knowledge, the coating of AlB_2_ with PFA has been implemented for the first time.

In this work, the AlB_2_ colloid was modified through the addition of specified amounts of HTPFA and poly(1,6-diisocyanatohexane) (N_100_) curing agent, utilizing a one-step in situ polymerization method. The morphology, thermal characteristics, ignition, and combustion characteristics of coated AlB_2_ were examined and compared with those of AlB_2_. In addition, the function of the HTPFA polymers in the combustion of AlB_2_ was clarified, which displayed guidance to utilize AlB_2_@HTPFA as a new high-energy fuel in energetic materials.

## 2. Materials and Methods

### 2.1. Materials

The raw AlB_2_ with a median particle size of 7.9 μm was supplied from Tangshan Weihao Magnesium Powder Co., Ltd. (Qian’an City, China) and manufactured by sintering technique before being used. The impurities were mostly unreacted with Al. N_100_ was brought from Shanghai McLean Biochemical Science and Technology Co., Ltd. (Shanghai, China). HTPFA was provided by Jiangsu Aikang Biomedical Research and Development Co., Ltd. (Nanjing, China). Butyl dilaurate (T_12_) with a purity of 95% as catalyst was acquired from Beijing Tongguang Fine Chemical Company (Beijing, China). The acetone used as the dispersion of N_100_ with an HPLC purity of 99.7% was provided by Beijing Tongguang Fine Chemical Company. The potassium perchlorate (KClO_4_) with a purity of 99.99%, employed as an oxidizer for laser ignition, was provided by Aladdin Company. For the laser ignition tests, KClO_4_ was selected as the oxidizing agent since it was a significant oxidant employed in the development of composites with impact safety [44,45].

### 2.2. Sample Preparation

To investigate the effect of HTPFA content on the properties of AlB_2_, three types of coated AlB_2_ with HTPFA mass ratios of 5 wt%, 10 wt%, and 15 wt% were prepared, respectively. The detailed preparation steps are as follows.

Firstly, a specific amount of N_100_ was added into the mixture of HTPFA and AlB_2_, which had been dissolved in the acetone under magnetic stirring. Secondly, a specific amount of T_12_ was added dropwise, which was followed by further magnetic stirring for three hours at room temperature. After filtration, washing, and drying at 55 °C for three hours, three different types of coated AlB_2_ were eventually obtained due to the reaction generating carbamate between the hydroxyl group on HTPFA and the isocyanate on N_100_. AlB_2_@HTPFA-5, AlB_2_@HTPFA-10, and AlB_2_@HTPFA-15 were the corresponding notations. The mass ratio of the two components of the composites AlB_2_@HTPFA was calculated using simulations of complete oxidation reactions, and the composites utilized in the laser ignition tests were made by physically combining for 30 min, 50 wt% of AlB_2_@HTPFA, and 50 wt% of KClO_4_.

### 2.3. Characterizations

A scanning electron microscope (SEM, SU8020, Hitachi, Tokyo, Japan) and an energy dispersive spectrometer (EDS) were used to analyze the morphology and elemental distribution of AlB_2_ and AlB_2_@HTPFA. The particle size distribution of AlB_2_@HTPFA was analyzed by a laser particle size analyzer (Malvern Mastersizer 2000, Malvern Panalytical, Malvern, UK). The crystalline phase of AlB_2_ and AlB_2_@HTPFA was analyzed by X-ray diffraction (XRD, D8 ADVANCE, BRUCKER, Karlsruhe, Germany) at 40 kV/40 mA, with a 2θ range of 5–90° in the pace of 0.02°/0.1 s. AlB_2_ and AlB_2_@HTPFA were subjected to XPS experiments using X-ray photoelectron spectroscopy (XPS, Escalab 250Xi, Thermo, Waltham, MA, USA).

Thermogravimetric analysis (TGA) and differential scanning calorimetry (DSC, STA449F3, NETZSCH, Selb, Germany) were simultaneously conducted to investigate the thermal reaction behaviors of the AlB_2_ and AlB_2_ @HTPFA. The sample of about 3 mg was heated from 25 °C to 1200 °C in an air atmosphere (50 mL/min) at a heating rate of 10 °C/min. The samples were analyzed using Bruker VERTEX 70 (Rosenheim, Germany) and Netzsch STA 449 F3 (Selb, Germany)-coupled thermogravimetric-Fourier transform infrared spectroscopy (TG-FTIR) analysis in which the sample of about 3 mg was heated from room temperature to 1000 °C in air atmosphere (50 mL/min) with a heating rate of 10 °C/min, while the FTIR spectral analysis ran. The scanning range of the FTIR detector was 4000~400 cm^−1^ with a resolution of 4 cm^−1^. A microcomputer automated calorimeter (MAC, TRHW-7000C, Hebi Tianrun Electronic Technology Co., Ltd., Hebi, China) was used to determine the calorific value of AlB_2_ and AlB_2_ @HTPFA. In this experiment, approximately 0.2 g of the samples was put into the calorimeter inflated with oxygen under 3 MPa, and the heat release was measured.

A constant volume combustion cell test of 30 mg of samples was conducted by the flame ignition method with an oxygen atmosphere (~3 MPa) to characterize the energy response and work capacity of the AlB_2_ and AlB_2_@HTPFA. The flame was generated by the tip of a nichrome wire, which was heated by a controlled DC current. The sensor recorded the changes in pressure during the vigorous oxidation process with time, and the measurements were repeated three times for each sample and averaged. Laser ignition experiments were used to assess the ignition combustion process of AlB_2_ and AlB_2_@HTPFA. The samples were ignited by a carbon dioxide laser (Shanghai Yuhong Laser Equipment Co., Ltd., Shanghai, China), and the flame was filmed at 8000 fps using a high-speed video camera (X113). The ignition power and ignition time were adjusted to 50 W and 500 ms, respectively. To study the combustion process of the particles using high-speed pictures, 30 mg of mechanically mixed samples was fired in the air and placed in the center of the ignition table.

## 3. Results and Discussion

### 3.1. Characterization of Morphology and Components

**Figure 1:**
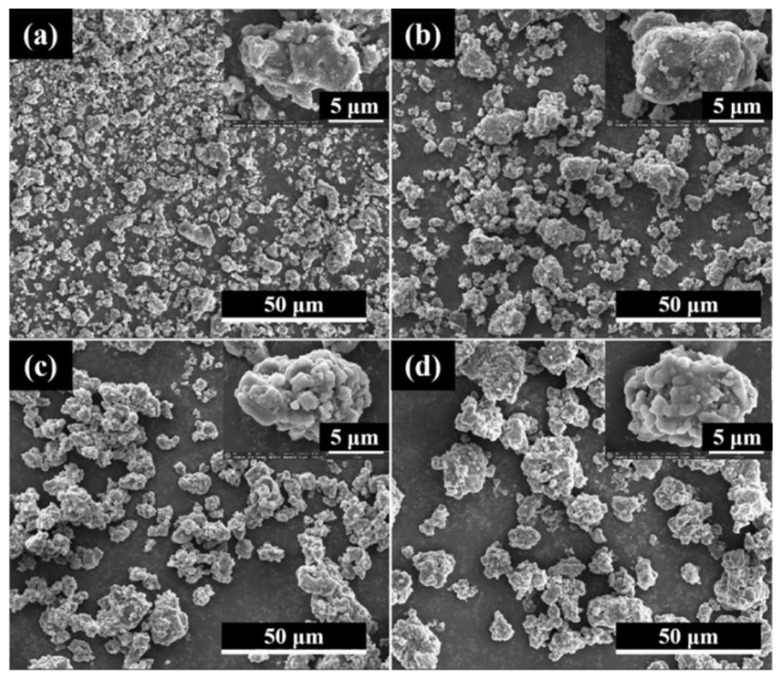
SEM images of (**a**) AlB_2_, (**b**) AlB_2_@HTPFA-5, (**c**) AlB_2_@HTPFA-10, and (**d**) AlB_2_@HTPFA-15.

The microstructure of AlB_2_ and AlB_2_@HTPFA was examined by SEM, and the morphology was shown in Figure 1. The majority of the raw AlB_2_ was irregular particles, while the AlB_2_@HTPFA particles were equally dispersed with no visible agglomeration. In comparison to the raw AlB_2_, as the content of the surface coating increased, the surface of AlB_2_@HTPFA gradually became smooth, demonstrating that the surface morphology of AlB_2_ might be altered by controlling the HTPFA polymer content.

**Figure 2:**
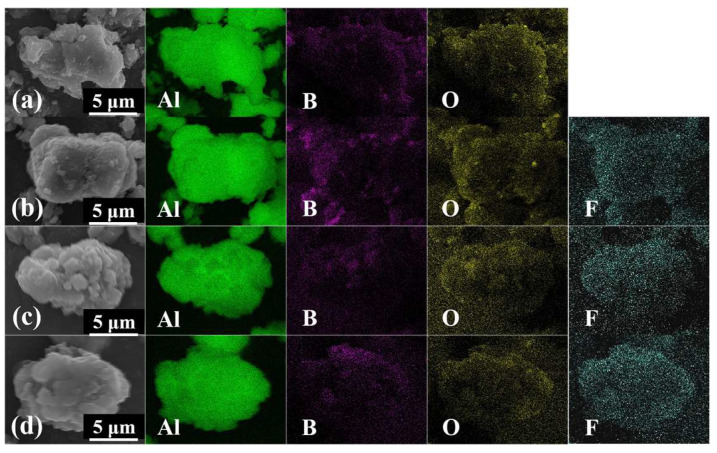
EDS images of (**a**) AlB_2_, (**b**) AlB_2_@HTPFA-5, (**c**) AlB_2_@HTPFA-10, and (**d**) AlB_2_@HTPFA-15.

The EDS results in Figure 2a indicated that the Al, B, and O elements were distributed evenly on the surface of AlB_2_, confirming the presence of oxide shells. In addition to Al, B, and O, the surface of the AlB_2_@HTPFA in Figure 2b–d was uniformly distributed with F elements, verifying that the HTPFA polymer was uniformly distributed on the surface of AlB_2_, proving the feasibility of the preparation method. The quantitative analysis results from EDS have been added to the Supporting Information (Table S1). The data reveal a gradual increase in surface F content with higher coating ratios, which conclusively confirms the successful coating of AlB_2_ by HTPFA.

**Figure 3:**
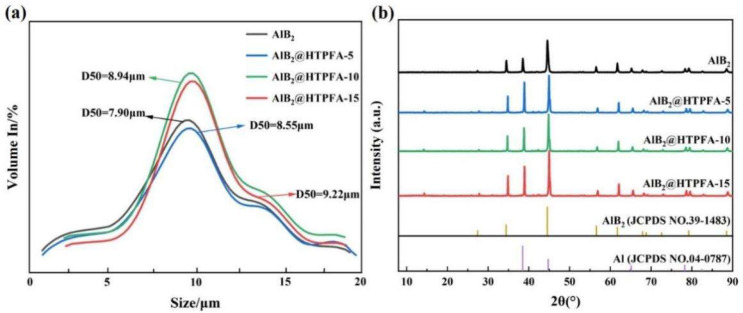
Particle size distribution curve (**a**) and XRD patterns (**b**) of AlB_2_ and AlB_2_@HTPFA.

Additionally, a laser particle size analyzer was used to characterize the particle size distribution of AlB_2_@HTPFA. The particle sizes of AlB_2_@HTPFA samples were regularly distributed, as illustrated in Figure 3a, and there was no evident agglomeration. The median diameters of the AlB_2_@HTPFA particles were 8.55 μm, 8.94 μm, and 9.22 μm, respectively, which is larger than that of the pristine AlB_2_ (7.9 μm), which demonstrates the feasibility of comparing the performances of AlB_2_ and AlB_2_@HTPFA. The XRD patterns of AlB_2_ and AlB_2_@HTPFA in Figure 3b revealed that the raw AlB_2_ was composed of Al and AlB_2_, and the presence of oxide shells was not detected since the oxide shell is amorphous. The crystalline composition of AlB_2_@HTPFA was identical to that of the AlB_2_, indicating that there was no change in the crystal structure of AlB_2_ during the preparation of the AlB_2_@HTPFA samples. There is no appearance of HTPFA patterns due to its amorphous form.

**Figure 4:**
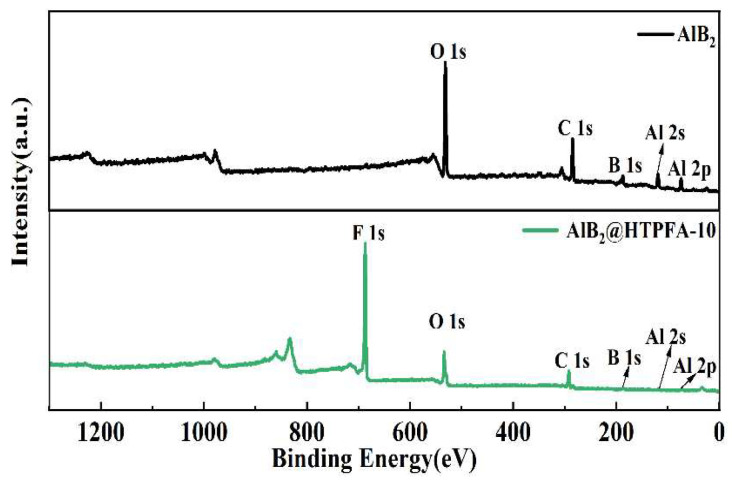
XPS spectra of AlB_2_ and AlB_2_@HTPFA-10.

To clarify the adsorption form of polymers on the surface of AlB_2_, an XPS of AlB_2_@HTPFA-10 was conducted [46]. As shown in Figure 4, the interface contact form between AlB_2_ and HTPFA was characterized, and the Al 2s, O 1s, C 1s, and F 1s peaks can be observed, which indicates the successful coating of HTPFA on AlB_2_ for AlB_2_@HTPFA-10.

**Figure 5:**
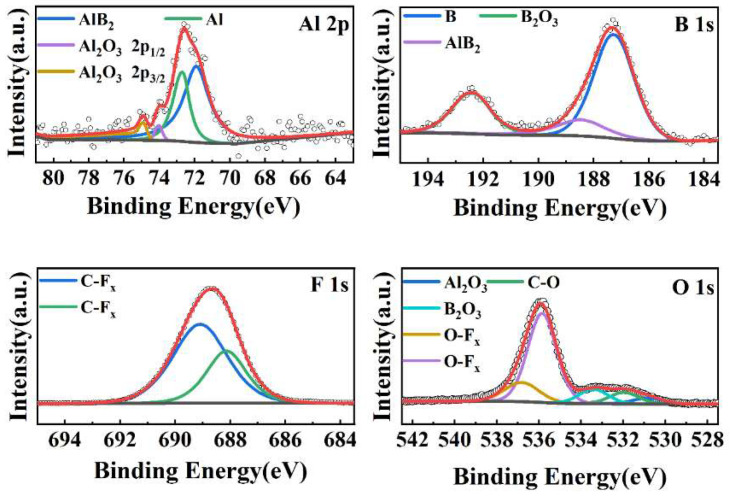
XPS spectra of Al 2p peaks, B 1s peaks, F 1s peaks, and O 1s peaks for AlB_2_@HTPFA-10.

The chemical states of Al, B, O, and F in AlB_2_@HTPFA-10 were investigated using split-peak fitting (Figure 5), which revealed C-O bonds (531.8 eV) and two O-F bonds (535.7 eV) in the O 1s peaks (536.8 eV), and two peaks (688.15 eV) with C-F bonds (689.1 eV) in the F 1s spectra, originated from HTPFA. There were no new Al-F or B-F bonds formed, indicating that the polymer was physically adsorbed on the surface of AlB_2_.

The XRD results of pristine AlB_2_ (Figure 3b) show diffraction peaks corresponding only to crystalline Al (PDF#04-0787) and AlB_2_ (PDF#65-2351), with no detectable diffraction peaks for aluminum oxide or boron oxide. This confirms the amorphous nature of the oxide shell (e.g., Al_2_O_3_ and B_2_O_3_), as XRD is only sensitive to crystalline phases. The XPS B 1s spectrum (Figure 5) reveals a weak peak at 187.5 eV, attributed to B-O bonds, indicating trace surface oxidation. However, the oxide layer thickness is insufficient to form a crystalline phase. After HTPFA coating, the intensity of the B-O peak decreases significantly, demonstrating the coating’s ability to suppress oxidation.

### 3.2. Oxidation Process

**Figure 6:**
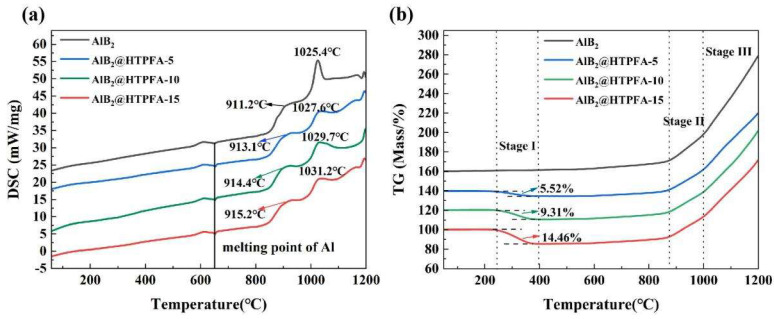
DSC/TG curves of AlB_2_ and AlB_2_@HTPFA samples: (**a**) DSC (**b**) TG.

TGA and DSC were used to investigate the oxidation process of AlB_2_ and AlB_2_@HTPFA in air. The DSC and TG curves shown in Figure 6 display that the reaction process of the AlB_2_@HTPFA samples can be divided into three stages based on mass changes: the decomposition of polymer (stage I), the initial oxidation of AlB_2_ (stage II), and the AlB_2_ decomposition oxidation reaction (stage III). There was no first-stage response observed in the curves of AlB_2_. The TGA/DSC curves of HTPFA have been added to the Supporting Information (Figure S1), and the TGA/DSC parameters have been quantified in the table (Table S2).

The decomposition of the polymer ranged from 25 °C to 400 °C. The weight loss of AlB_2_@HTPFA samples could be observed between 230 °C and 400 °C, indicating that polymer decomposition on the surface of the samples occurred at this temperature, and the mass loss was positively correlated with the ratio of the polymer shell. As the temperature increased, exothermic peaks developed in different samples at about 600 °C (Figure 6a), accompanied by a slow rise in mass due to the minor-quantity Al in the sample reacting with O_2_ and producing Al_2_O_3_ (Equation (1)) [47,48]. Al melted endothermically at about 660 °C, as seen by the endothermic peak in Figure 6a. The initial oxidation of AlB_2_ and AlB_2_@HTPFA occurred between 880 °C and 1000 °C when AlB_2_ interacted exothermically with O_2_ to produce Al_2_O_3_ and B_2_O_3_ (Equation (3)), and simultaneously amorphous alumina first changed into γ-Al_2_O_3_ [49]. Compared with amorphous alumina, the γ-Al_2_O_3_ owned greater density, which created more voids in the oxide shell, increasing the oxygen diffusion rate and sample quality during this phase.

**Figure 7:**
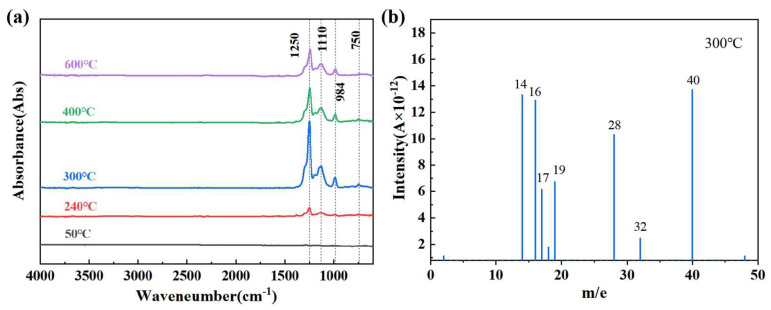
FTIR spectra of decomposition products of AlB_2_@HTPFA-10 at different temperatures (**a**) and MS spectra of decomposition products at 300 °C (**b**).

AlB_2_ decomposition oxidation reaction ranged from 1000 °C to 1200 °C. At high temperatures, AlB_2_ transformed into AlB_12_, which was influenced by parameters such as ball milling time and sintering time during the sintering preparation process of raw AlB_2_, corresponding to the conversion temperatures ranging from 956 ° C to 1350 °C reported by different researchers [50,51,52]. Endothermic peaks near 980 °C were observed in various samples (Figure 6b). Based on the temperature of AlB_12_ observed in Figure 7, the breakdown temperature of AlB_2_ in this investigation was near 980 °C. The decomposition process of AlB_2_ yielded a large amount of Al liquid, and, at higher temperatures, the presence of Al liquid could aid the oxidation process through rapid exothermic reactions and increased transport [48], immediately oxidizing on contact with air [53], resulting in a rapid increase in mass (Equation (4)).

To determine the decomposition products of AlB_2_@HTPFA in the first stage, the AlB_2_@HTPFA-10 was chosen for Fourier infrared testing with temperatures ranging from 25 °C to 600 °C. As the FTIR spectra of the products show in Figure 7a, the FTIR curve of the sample remained unchanged at 50 °C. At the initial decomposition temperature of 240 °C, significant absorption peaks could be observed around 1250 cm^−1^, corresponding to the bending vibration peaks of the C-F bond. Combined with the mass-to-charge ratio (m/e) of 19 in the mass spectrometry, which may correspond to F^−^, it indicates the presence of fluoride-containing compounds in the decomposition products. The appearance of these absorption peaks suggests that the polymer initially decomposes mainly into C_x_F_y_ during the initial stage of decomposition, which is consistent with the mass loss from 25 °C to 240 °C in the thermogravimetric (TG) curve shown in Figure 6b.

When the temperature is raised to 300 °C, three new distinct absorption peaks appear at 750 cm^−1^, 984 cm^−1^, and 1110 cm^−1^. Based on mass spectrometry analysis, they are, respectively, the symmetric vibration peak of the Al-F bond (750 cm^−1^) and the stretching vibration peaks of the C-O-C ether bond [36] (984 cm^−1^ and 1110 cm^−1^). Since no formation of Al-F or B-F bonds was found in the X-ray photoelectron spectroscopy (XPS) analysis of AlB_2_@HTPFA, the appearance of the Al-F bond at this time may be due to the reaction between HF decomposed from HTPFA and the Al_2_O_3_ on the surface. It is worth noting that HF was not detected in the infrared spectrum. This may be because the branched structure of HTPFA reduces the release of HF, resulting in most of the decomposition products existing in the form of C-F, and the small amount of HF generated reacts immediately with the oxides. As the temperature rises, the peaks of other substances remain basically unchanged, while the peaks of C_x_F_y_ first gradually weaken and then remain basically constant. This may be because after decomposition, some of the products react with the oxides on the surface of AlB_2_, reducing the content of F^−^. There are no other gas products that change significantly, indicating that the polymer decomposes completely at approximately 300 °C.

**Figure 8:**
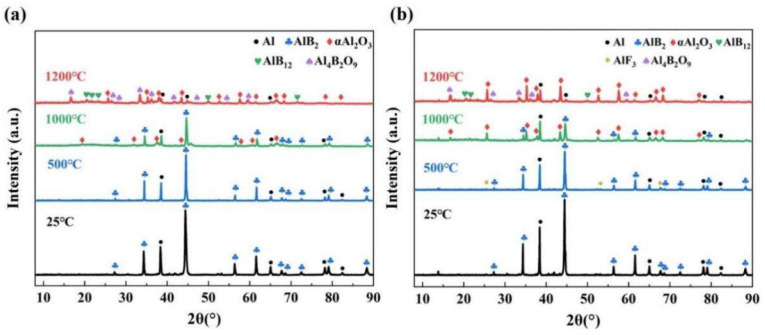
X-ray diffraction patterns of samples at different temperatures: (**a**) AlB_2_ and (**b**) AlB_2_@HTPFA-10.

Furthermore, to determine the intermediate products of AlB_2_ and modified AlB_2_ in the oxidation process, the reaction products of raw AlB_2_ and AlB_2_@HTPFA-10 samples at three different temperatures were characterized by XRD. Figure 8 exhibited that the diffraction peaks of AlB_2_ gradually weakened, and the peaks of α-Al_2_O_3_ began to appear in the process of the temperature approaching 1000 °C, indicating that the alumina underwent the crystalline phase transition in this process. It was suggested that in this process, the crystalline phase of alumina was changed from γ to α [47,49]. In addition, a small amount of unreacted Al remained in the product, and the AlF_3_ peaks were not identified in the AlB_2_@HTPFA at this temperature, owing to the small quantity of coating or the volatilization of some AlF_3_. As the reaction progressed, Al_2_O_3_ and B_2_O_3_ combined to form Al_4_B_2_O_9_ (2Al_2_O_3_·B_2_O_3_) at temperatures below 1035 °C, which decomposed to Al_18_B_4_O_33_ (9Al_2_O_3_·2B_2_O_3_) at higher temperatures [54,55], and the mass continued to grow. Although Al_18_B_4_O_33_ occurred at higher temperatures, it cooled and changed into Al_2_O_3_ and Al_18_B_4_O_33_ [56]; thus, the products were dominated by Al_2_O_3_ and Al_4_B_2_O_9_ with no B_2_O_3_, indicating that B_2_O_3_ was amorphous at this point.

The TGA curves of AlB_2_@HTPFA resembled that of AlB_2_. Aside from the conventional oxidation process of AlB_2_, it involved the reaction of surface fluoropolymer. The overall weight gain of AlB_2_, AlB_2_@HTPFA-5, AlB_2_@HTPFA-10, and AlB_2_@HTPFA-15 during the entire reaction phase was 118.63%, 80.76%, 80.47%, and 73.39%, respectively, indicating that certain compounds remaining on the surface of AlB_2_ were formed by the disintegration of the HTPFA coating layer at 400 °C. Because of the low permeability of these compounds, the oxygen transport on the surface of AlB_2_ was hindered. It indicated that some of the products produced by the decomposition of the HTPFA coating layer at 400 °C remained on the surface of AlB_2_. It was noted that the products showed poor breathability, which prevented the oxygen passage on the surface of AlB_2_. That was why the first and second oxidation peak temperatures of AlB_2_@HTPFA were more than 2 °C higher than those of AlB_2_.

Al + 0.75O_2_ → 0.5Al_2_O_3_, ∆H = −826.7 kJ/mol (1)

AlB_2_ + 2.25O_2_ → 0.5Al_2_O_3_ + B_2_O_3_, ∆H = −2241.1 kJ/mol (2)

AlB_2_ → 1/6AlB_12_ + 5/6Al, ∆H = 18.9 kJ/mol (3)

AlB_12_ + 9.75O_2_ → 0.5Al_2_O_3_ + 6B_2_O_3_, ∆H = −8727.1 kJ/mol (4)

Al_2_O_3_ + 0.5B_2_O_3_ → 0.5Al_4_B_2_O_9_, ∆H = −155.6 kJ/mol (5)

### 3.3. Calorimeter and Combustion Test

**Figure 9:**
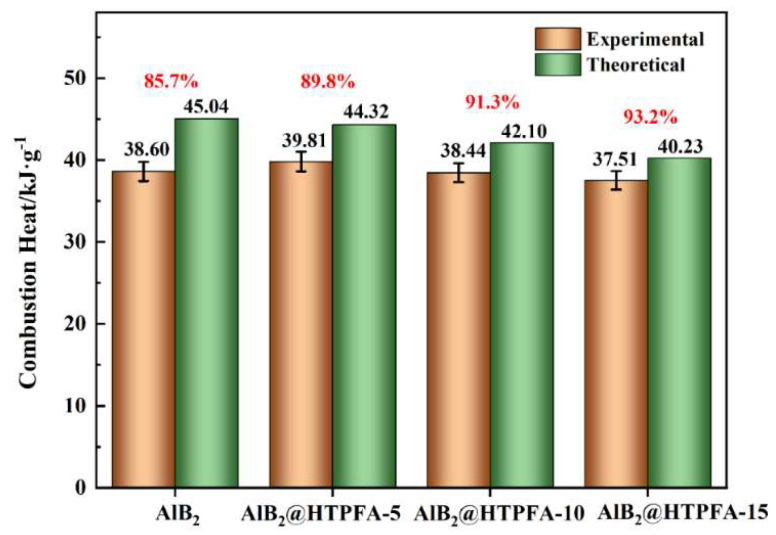
Test results of the calorific value and calculated energy efficiency.

To investigate the energy release of AlB_2_ and AlB_2_@HTPFA, the calorific value of samples was examined, and the findings are displayed in Figure 9. The heat of combustion increased initially and then decreased as the content of HTPFA increased. Due to the fact that Al produces more heat during the fluorination process (70.40 MJ/kg) compared with the oxidation process (36.29 MJ/kg) [57], AlB_2_@HTPFA-5 exhibited a higher calorific value than the basic raw AlB_2_. However, as the amount of the surface coating layer grows, the total calorific value drops due to the low calorific value of fluoropolymer. The results indicated that the HTPFA coating layer promoted the energy release of AlB_2_, and the energy-releasing rate steadily increased by 4.1%, 5.6%, and 7.5%, respectively, with the increasing HTPFA content. Therefore, it followed that exothermic fluorination between the surface oxide shells and fluorine stimulated the overall reactivity of AlB_2_, leading to a deeper degree of reactivity and improved energy efficiency of the modified AlB_2_@HTPFA.

The pressure change of AlB_2_ and AlB_2_@HTPFA with time was measured by the pressure cell. The rate of the pressure change was used to describe the vigorous oxidation process, which could be quantified as the rate of pressurization (MPa/ms), as shown in Equation (6).

Pressurization rate:

v = (P_max_ − P_i_)/(t_max_ − t_i_) (6)

where P_max_ represents the maximum pressure, P_i_ represents the pressure when the ignition process begins, t_max_ represents the time until the pressure reaches its maximum value, and t_i_ represents the ignition time.

**Figure 10:**
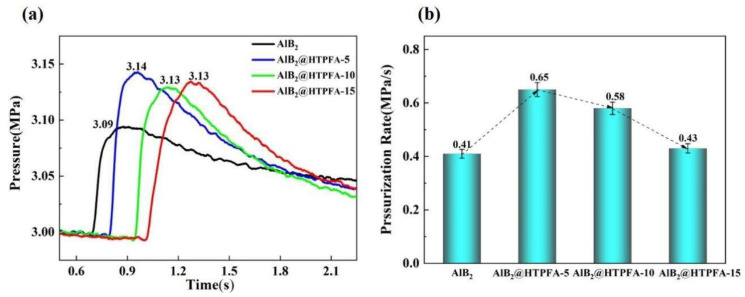
(**a**) The pressure changed with time and (**b**) the pressurization rate of AlB_2_ and AlB_2_@HTPFA.

The peak pressure of AlB_2_@HTPFA was higher than that of AlB_2_, as shown in Figure 10a. Figure 10b demonstrated that the pressurization rate of AlB_2_@HTPFA-5, AlB_2_@HTPFA-10, and AlB_2_@HTPFA-15 was all higher (0.65 MPa/s, 0.58 MPa/s, and 0.43 MPa/s, respectively) than that of AlB_2_ (0.41 MPa/s). It can be attributed to the fact that during the ignition process, the surface coating layer broke down quickly and reacted with the surface oxides of AlB_2_, causing an AlB_2_ preignition reaction (PIR) to occur before oxidation, promoting the contact between AlB_2_ and O_2_, deepening the reaction degree and speeding up the reaction rate.

### 3.4. Laser Ignition Measurement

**Figure 11:**
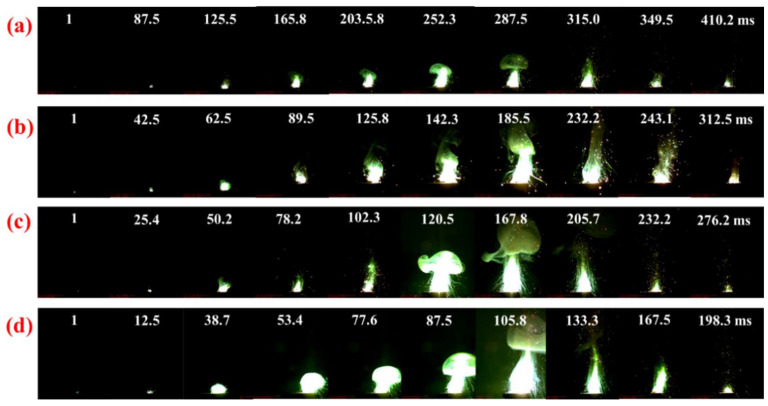
Ignition process of samples: (**a**) AlB_2_/KP, (**b**) AlB_2_@HTPFA-5/KP, (**c**) AlB_2_@HTPFA-10/KP, (**d**) AlB_2_@HTPFA-15/KP.

The ignition and combustion processes of energetic materials with KP as the oxidant were characterized by a laser ignition test with results depicted in Figure 11. AlB_2_/KP has a combustion time of 410.2 ms. The AlB_2_@KP burned for a shorter period of time after adding the HTPFA. Compared with AlB_2_/KP, AlB_2_@HTPFA-5/KP, AlB_2_@HTPFA-10/KP, and AlB_2_@HTPFA-15/KP exhibited reductions of 97.7 ms, 134.0 ms, and 211.9 ms, respectively. AlB_2_@HTPFA-15/KP reached the maximum flame with approximately 105.8 ms, which was earlier than AlB_2_@HTPFA-5/KP and produced more gaseous products, for the reason that as the content of the fluorine polymer on the surface increased, the decomposition of the fluorine polymer and oxidant produced a significant number of gaseous products in addition to the pre-ignition reaction between the oxide and fluorine polymer on the AlB_2_ surface. The diffusion and reaction processes involving AlB_2_ particles were accelerated. As a result, coating AlB_2_ with HTPFA polymer could improve the reactivity and reduce the combustion duration of AlB_2_-based energy materials.

It was crucial to note that laser ignition combustion test findings were different from those of combustion in a confined environment. The former was performed with the condition of adding oxygen in a restricted region, and the later was achieved with the condition of adding oxidant in an open area. It was found that during the ignition of AlB_2_@HTPFA, the confined region might prevent the diffusion of AlB_2_ particles. A portion of the poorly permeabilized decomposition products would stay on the AlB_2_ surface and impede the burning of AlB_2_ particles, causing the decrease in reactivity with the increasing coating concentration.

**Figure 12:**
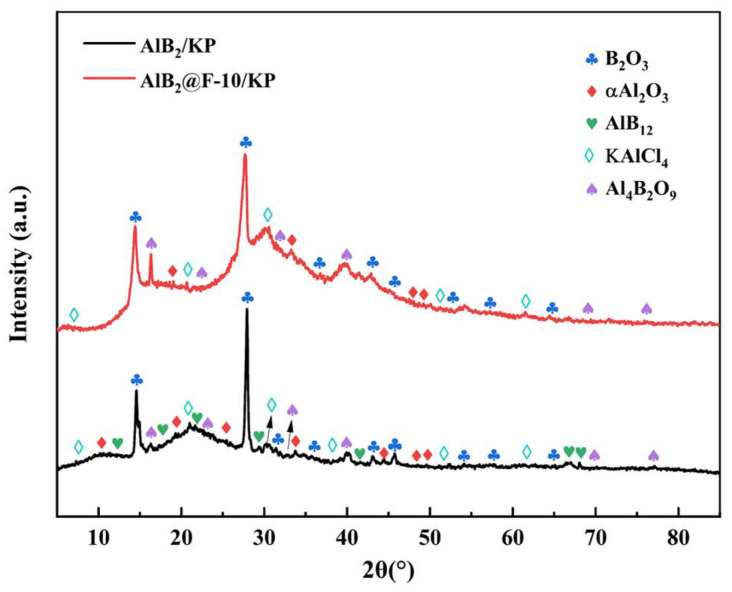
X-ray diffraction patterns of AlB_2_/KP and AlB_2_@HTPFA-10/KP.

To investigate the effect of AlB_2_@HTPFA on the combustion process further, the products were collected and studied by XRD (Figure 12). It was revealed that the products were predominantly composed of Al_2_O_3_, B_2_O_3_, KAlCl_4_, and Al_4_B_2_O_9_. It was found that AlB_12_ was present in the product of AlB_2_/KP due to the existence of HTPFA, while AlB_12_ was absent from the product of AlB_2_@HTPFA-10/KP. AlB_12_ was an intermediate product of AlB_2_ in the high-temperature oxidation process, indicating that mixed with KP, the reaction of AlB_2_@HTPFA in the rapid ignition process was more complete than that of AlB_2_.

Consequently, the ignition and combustion enhancement mechanism of AlB_2_ coated with HTPFA was proposed. It could be concluded that during the burning of AlB_2_@HTPFA-10/KP powder, the HTPFA on the surface broke down and reacted quickly, and the gas products’ HF reacted with the oxide shell on the surface of AlB_2_, generating a significant quantity of gas [6,58]. A pre-ignition reaction was obtained in this stage. The HF gas encouraged the diffusion of AlB_2_ particles and the interaction between AlB_2_ and KP, which could accelerate the combustion reaction of the composite fuel. As shown in Figure 11, high contents of HTPFA in AlB_2_@HTPFA-10/KP are beneficial for enhancing combustion rates. Additionally, the high combustion performance of AlB_2_ was obtained by adding the functionalized fluoropolymer. That was of great significance to designing and fabricating AlB_2_@functionalized fluoropolymer towards the potential application in high-energy propellant.

## 4. Conclusions

In this study, AlB_2_ coated with different contents (5 wt%, 10 wt%, and 15 wt%) of the HTPFA polymer was prepared. According to SEM, EDS, and particle size distribution analyses, the F element distributed uniformly on the surface of AlB_2_@HTPFA, and the particles dispersed evenly without noticeable agglomeration. The results of XRD and XPS analysis demonstrated that HTPFA polymer adheres to the surface of AlB_2_ particles. According to the thermal investigation, the oxidation peak temperature of samples increased as the amount of fluorine polymer increased. The results of combustion heat release indicated that AlB_2_@HTPFA-5 releases more energy than AlB_2_, and the combustion efficiency is increased by 4.1%. Meanwhile, the reactivity of AlB_2_@HTPFA-5 was 0.65 MPa/s, which is ~1.5 times that of AlB_2_. The ignition measurement illustrated that the pre-ignition interaction of the HTPFA polymer with surface oxide could accelerate the reaction and promote combustion efficiency. As a result, coating AlB_2_ with HTPFA polymer is a potential approach for regulating the ignition and combustion characteristics of AlB_2_ fuel, providing a viable way for the further application of AlB_2_ as a fuel in energetic materials.

## Data Availability

The original contributions presented in this study are included in the article. Further inquiries can be directed to the corresponding author.

## Supplementary Materials

The following supporting information can be downloaded at: https://www.mdpi.com/article/10.3390/ma18071452/s1, Table S1: EDS results; Figure S1: DSC/TG curves of HTPFA (Heating rate: 10 °C/min under air atmosphere); Table S2: Thermodynamic Parameters of Samples; Table S3: Compatibility of AlB_2_@HTPFA-10 with Contact Materials; Figure S2: First light times for the 9 different boron-PTFE mixtures are shown. Four experiments were conducted for each condition using 200 W/cm^2^; Figure S3: Images recorded from high speed video. Image A: PM1 (7.5 wt% PTFE); Image B: MM2 (15 wt% PTFE); Images C: PM3 (30 wt% PTFE); Image D: MM3 (30 wt% PTFE); Figure S4: Ignition process of samples: (a) AlB_2_/KP (b) AlB_2_@HTPFA-5/KP (c) AlB_2_@HTPFA-10/KP (d) AlB_2_@HTPFA-15/KP [30].
