# Supplementary material for: HTPFA-Coated AlB2 with Enhanced Combustion Performance as a High-Energy Fuel
Source: Materials (Basel). 2025 Mar 25;18(7):1452. doi: 10.3390/ma18071452 (PMC11989437; doi:10.3390/ma18071452)
Supplement: Supplementary file 1 [file materials-18-01452-s001.zip › materials-3518418-supplementary.pdf]

1. EDS Quantitative Analysis Data

Table S1. EDS results

Samples	Element	Weight%	Atomic%
AlB_2	Al	34.5	17.8
	B	60.8	78.2
	O	4.7	4.0
AlB_2@HTPFA-5	Al	37.8	19.6
	B	55.4	74.6
	O	6.4	5.5
	F	0.4	0.3
AlB_2@HTPFA-10	Al	34.8	18.2
	B	57.5	75.4
	O	6.9	5.8
	F	0.8	0.6
AlB_2@HTPFA-15	Al	37.1	19.2
	B	54.6	67.8
	O	6.9	6.0
	F	1.4	1.0

The data reveal a gradual increase in surface fluorine (F) content with higher coating ratios, which conclusively confirms the successful encapsulation of AlB_2 by HTPFA.

2. TGA/DSC Data and Thermodynamic Parameters of Samples

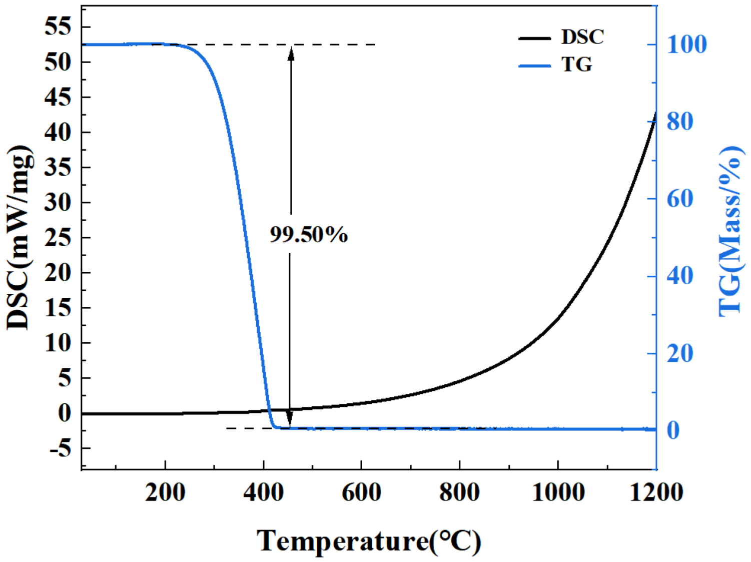

Figure S1. DSC/TG curves of HTPFA (Heating rate: 10 °C/min under air atmosphere**)**

Table S2. Thermodynamic Parameters of Samples

**Samples**	**First Oxidation Peak Temp. (or Initial Decomposition Temp.)/°C**	**Second Oxidation Peak Temp. (or Final Decomposition Temp.)/°C**	**Total Weight Gain/%**	**ΔH/(J·mg^{-1})**
HTPFA	230.2	401.3	-99.50	0.23
AlB_2	911.2	1029.8	118.63	30.46
AlB_2@HTPFA-5	913.1	1033.3	80.76	31.81
AlB_2@HTPFA-10	914.4	1035.4	80.47	26.87
AlB_2@HTPFA-15	915.2	1037.2	73.39	25.68

The data indicate that the thermal stability is slightly improved after HTPFA coating (the first oxidation peak temperature is delayed by approximately 2°C), which is likely due to the partial solid decomposition products of HTPFA remaining on the surface of AlB_2.

3 **Contact Material Compatibility Testing**

The compatibility between AlB_2@HTPFA and common contact materials in propellants was investigated by studying the outgassing amount. AlB_2@HTPFA-10 was selected as the sample. Each material was mixed with the sample at a mass mixing ratio of 1:1, and test specimens (approximately 5.0 g) were prepared. The test was conducted at 100.0°C for 48 hours. The results are shown in Table S3.

Table S3. Compatibility of AlB_2@HTPFA-10 with Contact Materials

Contact Material	Average Outgassing Volume V (mL)	Total Outgassing Volume VH (mL)	Net Outgassing Increase R (mL)	Conclusion
GAP	0.18	0.68	0.35	Compatible
HTPE	0.62	0.14	-0.63	Compatible
AP	0.18	0.12	-0.21	Compatible

Notes:Calculation formulas:V_{H2}=Vn-（V+V_{H1}），R=V_H-（V+V_{H2}）

Judgment criteria:

R<3.0mL: Compatible

R=3.0–5.0mL: Moderate reaction

R>5.0mL: Incompatible

4. Comparison of Ignition Characteristics

In the reference 1, Figure S2 and S3 demonstrate the ignition delay time and laser ignition images of material B compounded with varying contents of PTFE through ball milling. By comparing the ignition performance of the materials in this study under the same fluorine content conditions, it was found that the current system exhibited a more significant reduction in ignition delay time for the raw material, with an additional reduction of approximately 20% compared to the original system. This phenomenon may be attributed to the decomposition temperature of HTPFA (230°C), which is notably lower than that of PTFE (around 450°C). The difference in their thermal decomposition characteristics likely lowered the reaction activation energy, thereby accelerating the triggering of the ignition process.

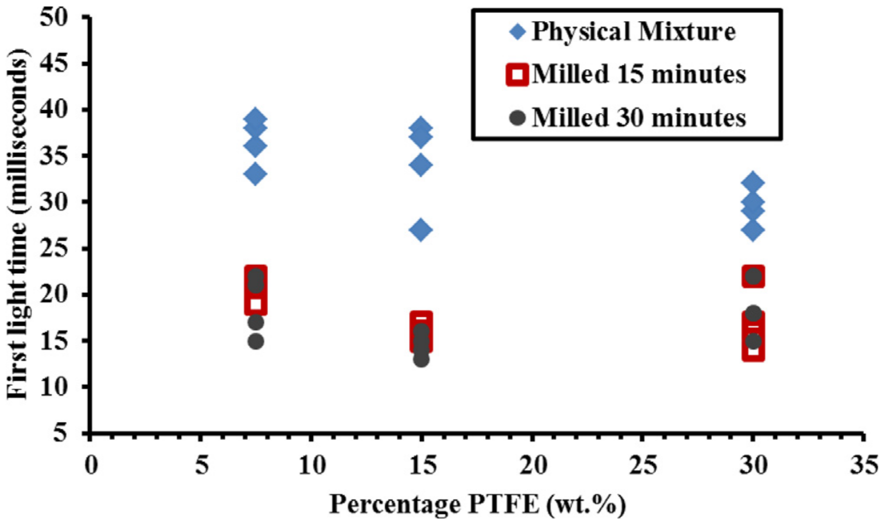

Figure S2. (First light times for the 9 different boron-PTFE mixtures are shown. Four experiments were conducted for each condition using 200 W/cm^2)

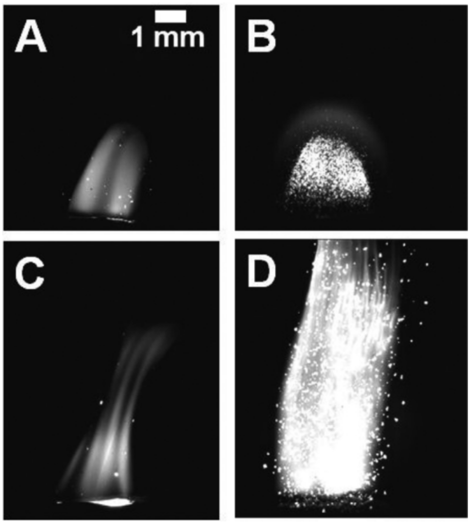

Figure S3. Images recorded from high speed video. Image A: PM1 (7.5wt.% PTFE); Image B: MM2 (15wt.% PTFE); Images C:PM3 (30wt.% PTFE); Image D: MM3 (30wt.% PTFE).

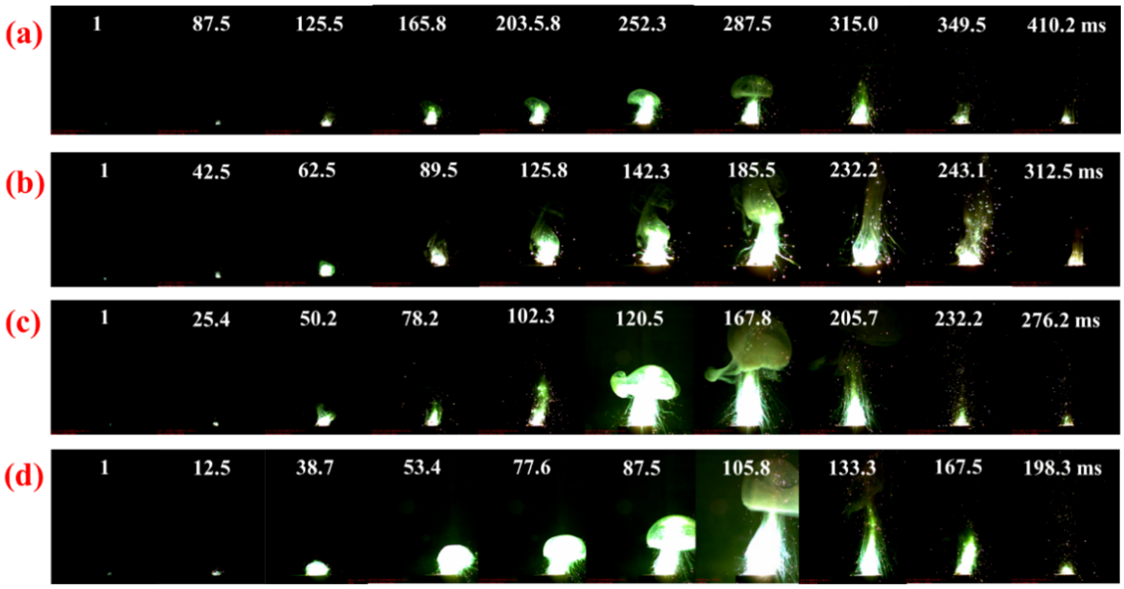

Figure S4. Ignition process of samples: (a)AlB_2/KP (b) AlB_2@HTPFA-5/KP (c) AlB_2@HTPFA-10/KP (d) AlB_2@HTPFA-15/KP

[1] Hedman T D , Demko A R , Kalman J .Enhanced ignition of milled boron-polytetrafluoroethylene mixtures.Combustion and Flame, 2018, 198(DEC.):112-119. DOI:10.1016/j.combustflame.2018.08.020.
